# Nasopharyngeal Carcinoma (NPC) Risk Factors: A Systematic Review and Meta-Analysis of the Association with Lifestyle, Diets, Socioeconomic and Sociodemographic in Asian Region

**DOI:** 10.31557/APJCP.2019.20.11.3505

**Published:** 2019

**Authors:** Simon I Okekpa, Rabiatul Basria S M N Mydin, Ernest Mangantig, Nor Syaffaf Amaliana Azmi, Siti Nur Syahirah Zahari, Gurjeet Kaur, Yusri Musa

**Affiliations:** 1 *Oncological and Radiological Sciences Cluster, Advanced Medical and Dental Institute, *; 3 *Regenerative Medicine Cluster, Advanced Medical and Dental Institute, Universiti Sains Malaysia, 13200 Bertam, Kepala Batas, Pulau Pinang, *; 4 *Universiti Teknologi MARA Cawangan Kelantan Kampus Kota Bharu, Wisma KUB, Jalan Sultan Ibrahim, 15050 Kota Bharu, Kelantan, *; 5 *Universiti Pendidikan Sultan Idris, 35900 Tanjong Malim, Perak Darul Ridzuan, *; 6 *Institute for Research in Molecular Medicine, Universiti Sains Malaysia,Malaysia, *; 2 *Department of Medical Laboratory Science, Faculty of Health Sciences, Ebonyi State University, Abakaliki, 840001 Ebonyi state, Nigeria. *

**Keywords:** nasopharyngeal carcinoma risk factors, dietary cancer risk in Asian, tobacco smoking

## Abstract

**Objective:**

Risk factors of nasopharyngeal carcinoma (NPC) have been linked with diets, life style and viral infections. NPC is more rampant in Asian populations than non-Asian countries. Our study aims to assess the validity of the suggestions provided by multiple case control studies demonstrating that salted fish consumption, smoking and alcohol consumption are associated with the risk of NPC in Asia.

**Methods:**

Search for related literature on salted fish, smoking and alcohol consumption were performed via Science Direct, PubMed databases and Google Scholar. Articles included in this study were from 2009 to 2017, with specific focus on salted fish, smoking and alcohol consumption as risk factors of NPC. This study excluded all articles published prior to 2009 and articles involving other cancers. Data were extracted independently by two different researchers and harmonized. Meta-analysis was conducted on the obtained data, by using R package Meta to create funnel and forest plots.

**Results:**

The meta-analysis revealed that salted fish, smoking and alcohol consumption were significantly associated to NPC risk with random effect model score showing OR of 1.41 at 95% confidence interval (CI) of 1.13-1.75 (P<0.01), OR of 1.89 at 95 % CI of 1.49 – 2.38, and OR: 1.42 at 95 % CI of 1.23 – 1.65 respectively. Our results also revealed significant association of salted meat, salted vegetables, house type, wood dust exposure associated with NPC risk with p values less than 0.05.

**Conclusion:**

This study proposes that salted fish intake, smoking and alcohol consumption might be linked to NPC risk in Asians. Further studies are necessary to ascertain the molecular mechanisms and clarify if the associated path that could function as therapeutic target.

## Introduction

Nasopharyngeal carcinoma (NPC) is more common in Asian than in non-Asian countries. In 2012, 86,691 cases (60,896 in males and 25,795 in females) of NPC and 50,831 NPC-related deaths (35,753 in males and 15,075 in females) were recorded globally. A total of 68,272 of the NPC cases were from Asia, in which majority of the cases were found in men (48,492 in men and 19,780 in women). The highest number of new NPC cases was from China (33,198), followed by Indonesia (13,084), Vietnam (4,931), India (3,947), and Malaysia (2,030) (Mahdavifar et al., 2016). NPC is highly prevalent among the Cantonese population, with an incidence of >20/100,000 in endemic areas (Zhang et al., 2015). In Southeast Asia, NPC incidence is high in Malaysia, Indonesia, Singapore, the Philippines, and Vietnam while low in Myanmar, Thailand, Lao republic, and Cambodia (Mahdavifar et al., 2016) The top five Asian countries with the highest age-standardized incidence rate of NPC were Malaysia (7.2 per 100,000), Singapore (6.4 per 100,000), Indonesia (5.6 per 100,000), Vietnam (5.5 per 100,000), and Brunei (4.6 per 100,000) (Mahdavifar et al., 2016). Meanwhile, the top five Asian countries with the highest age-standardized mortality rate were Indonesia (3.4 per 100,000), Vietnam (3.4 per 100,000), Singapore (2.8 per 100,000), Malaysia (2.5 per 100,000), and Brunei (2.1 per 100,000) (Mahdavifar et al., 2016). 

Several studies have linked lifestyle factors such as smoking and alcohol consumption and dietary factors such as consumption of preserved foods with NPC risk (Polesel et al., 2013; Lakhanpal et al., 2014; Ekpanyaskul et al., 2015; He et al., 2015; Lourembam et al., 2015; Xie et al., 2015; Ren et al., 2017; Yong et al., 2017). However, results across various studies have not been entirely consistent. The high incidence of NPC found among the Chinese population in China that migrated to other regions of the world suggests that the dietary habit of this population contributes to NPC development (Chattopadhyay et al., 2017). Due to economic growth and development over the last decade, lifestyle and dietary habits have changed. For example, traditional Chinese diets shifted to western diets (Lee et al., 2003; Cao et al., 2011; Carioli et al., 2017). Thus, modification of dietary habits and changes in environmental factors may have contributed to the recent decreasing trends of NPC in Hong Kong, Taiwan, and Singapore (Tsao et al., 2014). To date, a systematic review of available studies on the risk factors of NPC in Asia remains lacking. 

This study aims to provide an updated systematic review and meta-analysis on the daily activities such as consumption of salted fish, alcohol consumption, smoking, smoked meat consumption, sociodemographic factors, socioeconomic factors which could pose threat to human life in terms of their association with increased NPC risk in Asian population.

## Materials and Methods

This study was conducted according to the Preferred Reporting Items for Systematic Reviews and Meta-Analyses (PRISMA) statement.

*Search Strategy*

**Figure 1:**
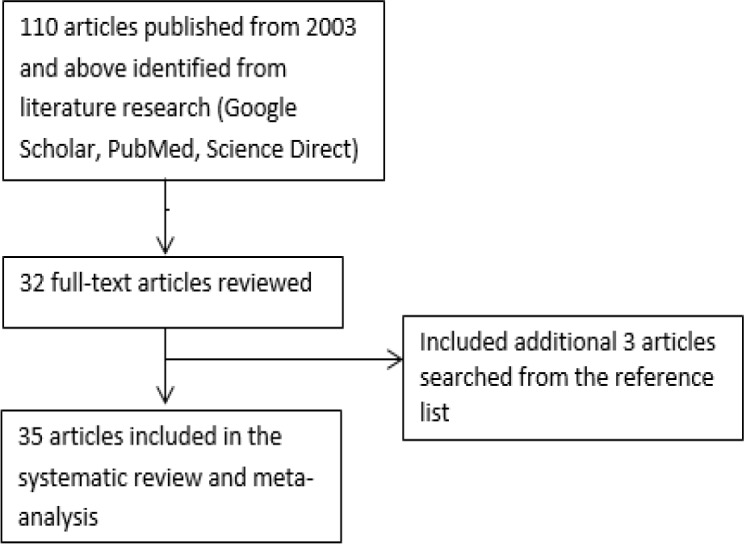
Identification of Relevant Studies for the Incidences, Mortalities and Risk Factors of NPC. Google Scholar, Science Direct, and PubMed databases were searched, and 110 related articles were identified which was screened and trimmed to 35 relevant publications

To identify relevant studies for the incidences, mortalities and risk factors of NPC, we searched the Google Scholar, Science Direct, and PubMed databases to identify the relevant publications (Figure 1). The keywords that were used to search for relevant articles are nasopharyngeal cancer, lifestyle, dietary, wood exposure, tobacco smoking, alcohol consumption, salted fish, preserved food, and the incidence of NPC. Also, we performed a manual search for references cited in the selected relevant articles and published reviews to search for additional relevant studies.

*Selection Criteria*

The selected studies were screened for eligibility and relevance. The criteria for inclusion in this systematic review are; all publications must have been published within the past 15 years (2003 till 2018), it must be papers that published only nasopharyngeal carcinoma cases, the paper must address the association of lifestyle, dietary, socioeconomic, and sociodemographic factors with NPC risk, and the studies must have case-control design. The exclusion criteria are all publications published before 2003, studies including another type of head and neck cancer (HNC) besides nasopharyngeal cancer and other cancer types, and studies not addressing the association of lifestyle, dietary, socioeconomic, and sociodemographic with NPC risk.

*Data Extraction*

The selected studies were reviewed critically. Data were extracted by two independent reviewers and cross-checked to reach consensus. All quantitative results for the association of lifestyle, dietary, socioeconomic, and sociodemographic factors with NPC risk were extracted, and the summary of the evidence for risk factors of NPC are shown in Table 1-6. The following variables from each study were recorded: the first author’s last name, publication year, number of cases and controls in the study, the country where the study was performed, data collection method, primary outcome measured in the study, other outcomes measured, odds ratio (OR) estimates with corresponding 95% confidence interval. 

*Statistical Analysis*

Meta-analysis was performed for smoking habit and alcohol consumption using the 15 case-control studies reporting association for smoking and 10 case-control studies reporting association for alcohol, respectively. The number of cases and controls from each selected study were used to compute odds ratio and were summarized using random-effect model for two risk factors (smoking habits and alcohol consumption) that were commonly reported in case-control studies. Statistical heterogeneity among the studies included in the meta-analysis was evaluated using Q and I^2^ statistics for the two risk factors. Forest plot was computed for smoking habit and alcohol consumption, respectively using R package meta. Estimation of potential publication bias was executed using funnel plot, in which the standard error of OR of each study was plotted against its OR. An asymmetrical plot suggests possible publication bias. All statistical analyses were performed using R Software version 3.4.3. A P-value <0.05 was considered statistically significant. 

**Table 1:** Summary of the Case-Control Studies on the Risk Factors Contributed to Nasopharyngeal Cancer

Sources	Participant, Study location	Data collection method	Outcome measured	Adjusted OR estimates (95% CI)
Yong et al., (2017)	290 cases and controls, Singapore	Questionnaire Interview	Salted fish consumption	Monthly: OR = 1.41(0.88 –2.26) Weekly/daily: OR = 4.18 (1.69-10.38)
Ren et al., (2017)	118 patients, 274 controls, China	Interviewed via telephone	salted fish consumption	Monthly: OR = 1.53 (0.85–2.73) Weekly: OR = 1.71 (0.93–3.17)
Ghosh et al., (2014)	64 cases, 100 controls, India	Personal interview	Intake of salted fish	OR_saltedfish_ = 2.61
Yong et al., (2017)	290 cases and controls, Singapore	Questionnaire, Interview	Salted meat	Monthly: OR = 2.04 (1.18 – 3.50) Weekly/daily: OR = 2.18 (0.97– 4.89)
Yong et al., (2017)	290 cases and controls, Singapore	Questionnaire Interview	Salted vegetable	Monthly: OR = 1.54 (0.99 – 2.39) Weekly/daily: OR = 3.70 (1.58 – 8.64)
Ghosh et al., (2014)	64 cases, 100 controls, India	Personal interview	Intake of smoked fish.	OR_smokedfish_ = 2.21
Yong et al., (2017)	290 cases and controls, Singapore	Questionnaire, Interview	Intake of smoked fish	Monthly: OR = 0.84 (0.47 – 1.50) Weekly/daily: OR = 1.33 (0.30 – 5.96)
Ghosh et al., (2014)	64 cases, 100 controls, India	Personal interview	Intake of smoked meat,	OR_smokemeat_ = 2.00
Yong et al., (2017)	290 cases and controls, Singapore	Questionnaire, Interview	Intake of smoked meat	Monthly: OR = 0.75 (0.47–1.18) Weekly/daily: OR = 1.52 (0.55– 4.24)
Yong et al., (2017)	290 cases and controls, Singapore	Questionnaire, Interview	Smoking (currently vs ever-smoke)	Current smokers: OR = 4.50 (2.58–7.86) Former smokers: OR = 2.52 (1.54–4.12)
Ren et al., (2017)	118 patients, 274 controls, China	Interviewed via telephone	Smoking	10-30 cigarettes: OR = 4.03 (1.11-14.68) < 30cigarettes: OR = 11.46 (1.26-103.91)
Xie et al., (2015)	352 cases, 410 controls, Hong Kong	Questionnaire	Smoking (currently vs ex-smoker)	Currently smoking: OR_adj_ = 1.67 (1.06-2.61) Ex-smoker: OR_adj _= 1.51 (0.94-2.41)
He et al., (2015)	1,845 cases, 2,275 controls, Guangdong, China	Interview	Wood combustion, cigarette smoking, and family history, Incense burning	Frequent incense use: OR = 1.73 (1.43-2.09) Wood fuel use: OR = 1.95 (1.65-2.31) Incense burning and cigarette synergistic index (SI): OR = 1.67 (1.01-2.76) Wood fuel use and family history SI: OR = 1.77 (1.06-2.96)
Lourembam et al., (2015)	105 cases, 115 controls, North-eastern India	Interview	Smoked meat consumption, exposure to smoke, living in house with poor ventilation, and alcohol consumption	OR (95% CI) was not determined in the study. Only p-values were reported: Smoked meat consumption = p<0.00001 Exposure to smoke = p<0.0007 Living in house with poor ventilation = p<0.0032 Alcohol consumption = p<0.01
Lakhanpal et al., (2014)	120 patients, 100 controls, India	Questionnaire Interview	Use of firewood, living in mud house, and consumption of alcohol	Use of fire wood: OR = 3.79 (1.97-7.30) Mud house: OR = 3.46 (1.19-10.08) Alcohol: OR = 2.11 (1.02-4.37)
Ekpanyaskul et al., (2015)	327 cases, 327 controls, Thailand	Personal interview	Wood dust exposure	OR = 1.62 (1.03-2.74)
Ren et al., (2017).	118 patients, 274 controls, China	Interviewed via telephone	High school or higher Education level	OR = 0.58 (0.32–1.05)

OR, Odds Ratio; CI, Confidence Interval.

**Table 2:** Case-Control Studies on the Association of NPC with Dietary Habits

Factors	Study	P-value
Salted fish	Yong et al, (2017)	0.033
	Liu et al., (2017)	0.02
	Xie et al., (2015)	0.199
	He et al., (2015)	<0.001
	Ghosh et al., (2014)	0.02
	Lakhanpal et al., (2014)	0.47
	Jia et al., (2010)	0.001
	Guo et al., (2009)	<0.001
Salted meat, Salted vegetables, Preserved vegetables & Smoked meat	Yong et al, (2017)	0.003, <0.001& 0.54, 0.06 respectively
	Lourembam et al., (2015)	<0.001
	Ghosh et al, (2014)	0.03
Intake of dark green vegetables	Xie et al., (2015)	0.001
	He et al., (2015)	<0.001
Habitual drinking of herbal tea	Xie et al., (2015)	0.012

**Table 3:** Case-Control Studies on NPC Association with Smoking Habit

Study	Cases group		Control group		OR (95%-CI)
	Cases	Total	Control	Total	
Xie et al., (2015)	101	352	69	410	1.99 (1.41-2.81)
Lourenbam et al., (2015).	56	105	54	115	1.29 (0.76-2.19)
He et al., (2015).	972	1,845	893	2,275	1.72 (1.52-1.95)
Yong et al., (2017).	144	290	78	290	2.68 (1.89-3.79)
Liu et al., (2017).	1,390	2,499	1,368	2,576	1.11 (0.99-1.24)
Ghosh et al., (2014).	43	64	27	100	5.54 (2.79-10.9)
Tsai et al., (2016).	73	176	150	352	0.95 (0.66-1.38)
Fachiroh et al., (2012).	404	681	407	1078	2.40 (1.98-2.93)
Hashim et al., (2012).	20	96	17	96	1.22 (0.60-2.51)
Ji et al., (2011).	516	1,044	312	1,095	2.45 (2.05-2.93)
Ekburanawat et al., (2010)	206	327	147	327	2.08 (1.50-2.85)
Guo et al., (2009)	522	1,049	396	785	0.97 (0.01-1.17)
Turkoz et al., (2011)	115	183	64	183	3.14 (2.05-4.82)
Cao et al., (2011).	298	462	158	511	4.06 (3.11-5.30)
Yang et al., (2005).	192	502	617	1,942	1.33 (1.08-1.63)

OR, Odds Ratio; CI, Confidence Interval

**Table 4:** Case-Control Studies on NPC Association with Alcohol Consumption

Study	Cases group		Control group		OR (95%-CI)
	Cases	Total	Control	Total	
Yong et al., (2017)	176	290	170	290	1.09 (0.78-1.52)
Tsai et al., (2016).	72	176	124	352	1.27 (0.88-1.85)
Lakhanpal et al., (2015)	69	120	39	100	2.12 (1.23-3.63)
Lourenbam et al., (2015)	59	105	45	115	2.00 (1.17-3.42)
Ghosh et al., (2014)	43	64	49	100	2.13 (1.11-4.09)
Fachiroh et al., (2012)	386	681	475	1078	1.66 (1.37-2.02)
Hashim et al., (2012)	10	96	10	96	1.00 (0.40-2.52)
Ji et al., (2011)	243	1,044	218	1,095	1.22 (0.99-1.50)
Ekburanawat et al., (2010)	198	327	181	327	1.24 (0.91-1.69)
Turkoz et al., (2011)	34	183	23	183	1.59 (0.89-2.82)

**Figure 2:**
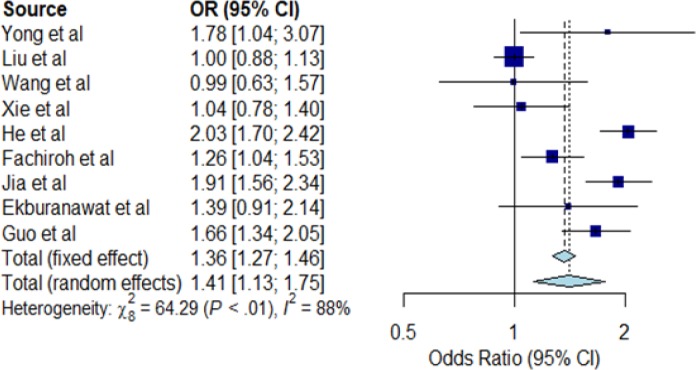
Forest Plot for Salted Fish Consumption and NPC Risk. The Individual horizontal line as seen on the forest plot is representing a single study with the blue box portraying the result plotted while the line shows the 95% confidence interval of the displayed result. The bigger the box, the larger the size of the study. The diamond shape at the lowermost part of the plot demonstrates the average of the combined result (1.41) of all the individual studies whereas the 95% confidence intervals (1.13; 1.75) is represented by the horizontal points of the diamond. The horizontal points of the diamond did not cross the vertical line, therefore our study detected statistically significant differences between the studies. I^2^ statistic provided an idea on heterogeneity (88%) of the studies, therefore showing inconsistency of the studies. Note that any I^2^ value of >50%, means inconsistent studies

**Table 5:** Case-Control Studies on the Association of NPC with Socioeconomic Factors

Factors	Study	Exposure	P-value
Occupational exposure	Hashim et al, 2012	No Yes	<0.001
	Guo et al., (2009)	<10 years ≥10 years	0.99 0.001
Dust exposure	Lourembam et al., (2015)	Ever	0.21
Type of household	Liu et al., (2017)	Building/concrete Cottage/clay brick.	<0.001
	He et al., (2015)	Block Bungalow	<0.001
	Xie et al., (2015)	Public rental housing estates Homeownership scheme courts Private mansion houses Private tenement houses	<0.001
	Lakhanpal et al., (2014)	Brick/concrete Mud Semi-brick/concrete	- 0.02 0.99
Poor ventilated house.	Lourembam et al., (2015)	Poor ventilated house	0.0032
Smoke Exposure	Lourembam et al., (2015)	Smoke Exposure	0.0007
Cooking experience at home	Xie et al., (2015)	Cooking fumes	0.005

**Figure 3:**
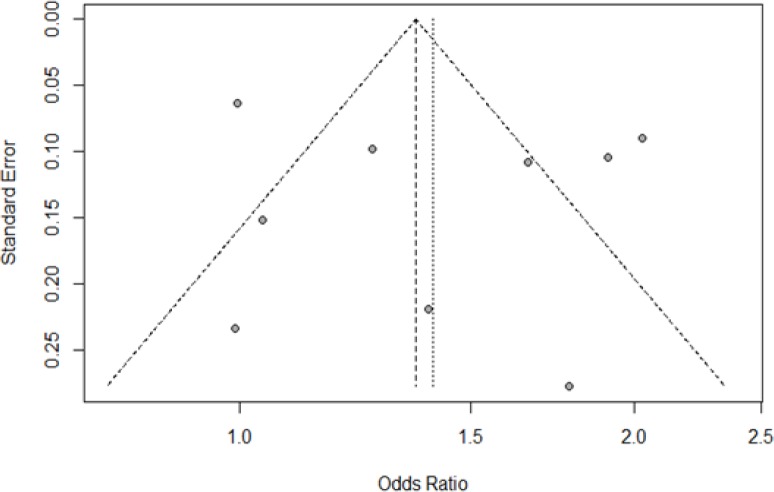
Funnel Plot for Salted Fish Consumption. Each dot signifies individual study. The standard error of the effect estimate is represented by y-axis. X-axis represents the result/odds ratio for the study. Towards the top are larger studies with higher power while toward the bottom are studies with lower power. The scatter in the plot is because of sampling variation. The shape and scatter of the plot is due to variety of standard errors in the studies. Assuming the studies have the same size of standard errors, all the studies/dots would have dropped on the horizontal line

**Table 6:** Case-Control Studies on the Association of NPC with Sociodemographic Factors

Factors	Study	Groups compared	p-value
Level of education	Xie et al., (2015)	None	<0.001
		Primary	
		Secondary	
	He et al., (2015)	Primary	0.63
		Secondary	
		College	
	Fachiroh et al., (2012)	≤12 years	<0.001
		>12 years	
	Jia et al., (2010).	None/Primary	0.99
		Secondary	
		High school.	

**Figure 4:**
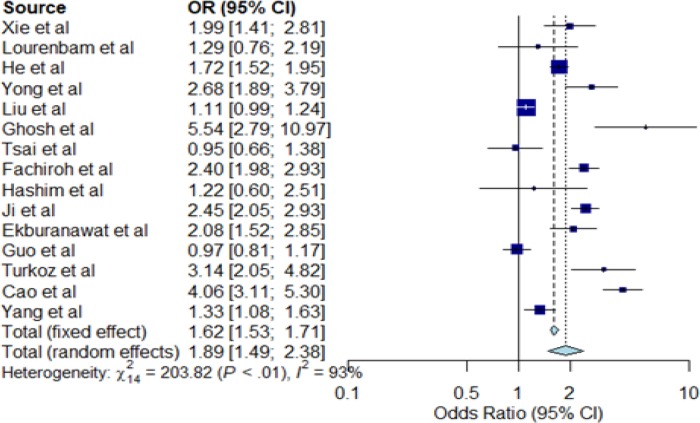
Forest Plot for Smoking Habit and NPC Risk. The Individual horizontal line as seen on the forest plot is representing a single study with the blue box portraying the result plotted while the line shows the 95% confidence interval of the displayed result. The bigger the box, the larger the size of the study. The diamond shape at the lowermost part of the plot demonstrates the average of the combined result (1.89) of all the individual studies whereas the 95% confidence intervals (1.49; 2.38) is represented by the horizontal points of the diamond. The horizontal points of the diamond did not cross the vertical line; therefore, our study detected a statistically significant difference between the studies. I2 statistic provided an idea on heterogeneity (93%) of the studies, therefore showing inconsistency of the studies. Note that any I2 value of >50%, means inconsistent studies

**Figure 5:**
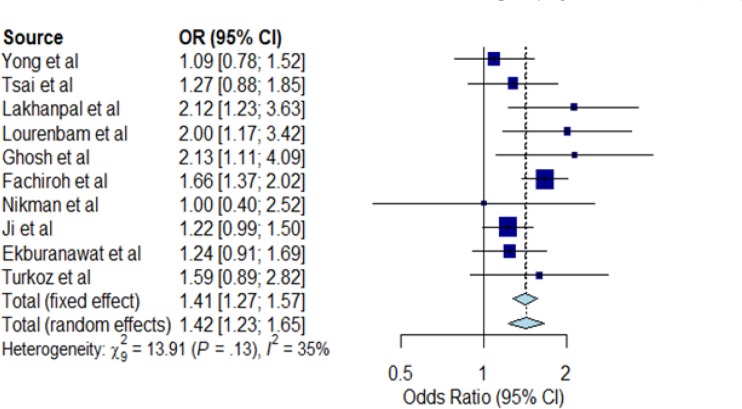
Forest Plot for Alcohol Consumption and NPC Risk. The Individual horizontal line as seen on the forest plot is representing a single study with the blue box portraying the result plotted while the line shows the 95% confidence interval of the displayed result. The diamond shape at the lowermost part of the plot demonstrates the average of the combined result (1.42) of all the individual studies whereas the 95% confidence intervals (1.23; 1.65) is represented by the horizontal points of the diamond. The horizontal points of the diamond did not cross the vertical line, therefore our study showed statistically significant difference between the studies. I2 statistic showed heterogeneity of 35% between the studies

**Figure 6.**
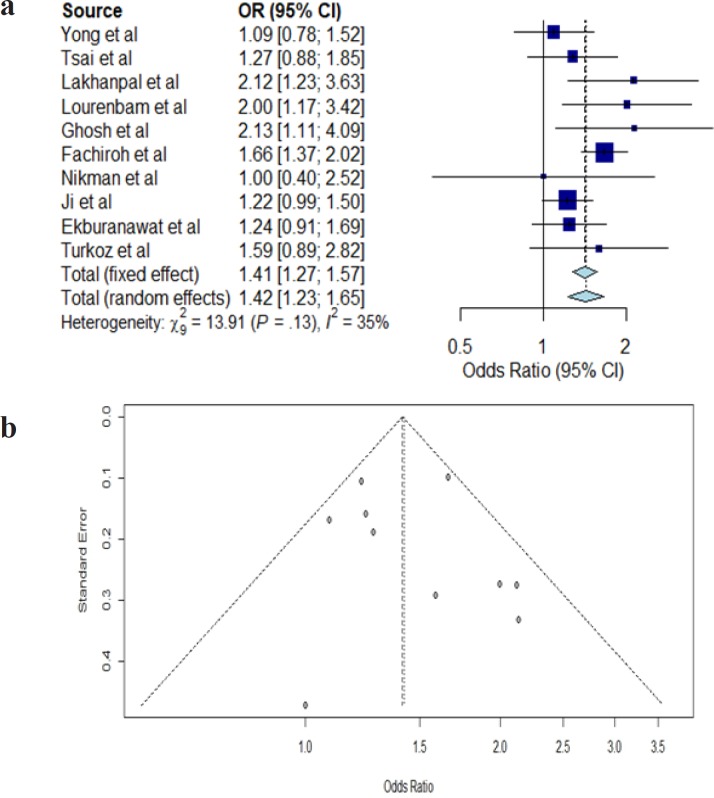
a, Funnel Plot for Smoking Habit. Each dot signifies individual study. The standard error of the effect estimate is represented by y-axis. X-axis represents the result/odds ratio for the study. Towards the top are larger studies with higher power while toward the bottom are studies with lower power. The scatter in the plot is because of sampling variation. The shape and scatter of the plot is due to variety of standard errors in the studies. Assuming the studies have the same size of standard errors, all the studies/dots would have dropped on the horizontal line; b, Funnel Plot for Alcohol Consumption. Each dot signifies individual study. The standard error of the effect estimate is represented by y-axis. X-axis represents the result/odds ratio for the study. Towards the top are larger studies with higher power while toward the bottom are studies with lower power. The scatter in the plot is because of sampling variation. The shape and scatter of the plot is due to variety of standard errors in the studies. Assuming the studies have the same size of standard errors, all the studies/dots would have dropped on the horizontal line

## Results

*Literature Search*

Figure 1 shows a flowchart of the search process for the studies included in this systematic review and meta-analysis. The characteristics of the selected studies are summarized in Tables 2, 3, 4 and 5. All of the articles included in this study were published between 2003 and 2017. Only studies with the case-control design were included in this review due to the lack of cohort studies on the risk factor of NPC.

During the literature search we were able to identify one hundred and ten (110) articles related to NPC risk that were published between 2003 and 2017. Thirty-two articles out of the 110 met the stipulated criteria for inclusion in our study while additional three relevant articles were discovered from the reference list of the chosen articles making it a total of thirty-five articles reviewed for this study. 

*Evidence for risk factors of NPC*

The primary studies on risk factors for NPC are listed in Table 1. Various methods were used to obtain information related to tobacco smoking habits, alcohol drinking, occupational exposure, consumption of salted fish, and other preserved foods. Due to differences in measurement of study factors and adjustment for other confounding factors, the meta-analysis was only conducted for smoking habit, alcohol consumption and consumption of salted fish.

*Dietary factors*

Several case-control studies evaluated the association of dietary pattern which refers to intake of salted fish, Salted meat, Salted vegetables, smoked meat, Intake of dark green vegetables, Habitual drinking of herbal tea and preserved vegetables with NPC risk as shown in Table 2. Due to different measurement strategy of the exposure pattern in each study, it was not possible to conduct a meta-analysis to obtain a summary OR for the association of smoked meat, Salted vegetables, Salted meat, Habitual drinking of herbal tea, Intake of dark green vegetables and preserved vegetables consumption with NPC risk. Our result revealed that six out of eight studies that reported the association of Salted fish with NPC were significantly associated with NPC risk. Of the three studies that reported the association between smoked meat consumption and NPC risk, two were found to have significant association. For the preserved vegetables consumption and NPC risk, the only one study reported has statistically nonsignificant association. Salted meat and salted vegetables were all significantly associated with NPC risk from the reported data. Two reported data on the intake of dark green vegetables all suggested significant association with NPC.

*Meta-analysis for salted fish consumption*

The meta-analysis for salted fish consumption (ever consumed versus never consumed) are shown in Figure 2. The association between salted fish consumption and risk of NPC was statistically significant in the random effect model (OR=1.41; 95% CI=1.13-1.75) but with a large statistical heterogeneity (I^2^=88%; P<0.01) between the nine case-control studies included in the meta-analysis. Based on the funnel plot, there is no visual indication of publication bias Figure 3.

*Lifestyle factors*

Fifteen case-control studies that examined the association between smoking habits and NPC risk in Asia were studied (Table 3). Of the 15 studies, only ten (66.7%) reported that smoking significantly increased the risk for developing NPC. The meta-analysis result obtained from our forest plot in Figure 4 show significant (OR=1.89; 95% CI=1.49-2.38) association of NPC risk with smoking, but with large heterogeneity (I^2^=93%; P<0.01) as shown in Figure 6a. 

The association between alcohol consumption and NPC risk in Asia were assessed from ten case-control studies as shown in Table 4. Only four out of the ten studies reported alcohol significantly increased the risk for NPC. The meta-analysis result obtained from our forest plot in Figure 5 show significant (OR=1.42, 95% CI=1.23-1.65) association of NPC risk with alcohol consumption but with low heterogeneity (I^2^=35%, P=0.13) as shown in Figure 6b. 

*Meta-analysis for smoking and alcohol consumption*

The results of the meta-analyses for smoking (smoker versus non-smoker) and alcohol consumption (alcohol drinker versus non-drinker) are shown in Figure 4 and 5, respectively. In meta-analysis, the association between smoking and risk of NPC was statistically significant in the random effect model (OR=1.89; 95% CI=1.49-2.38) shown in Figure 4. A large statistical heterogeneity shown among the 15 case-control studies included in the meta-analysis (I^2^=93%; P<0.01). As the case-control studies have different study characteristics and study factors, we could not conduct further meta-regression analysis to investigate other factors that could potentially influence the summary odds ratio for NPC risk. Based on the funnel plot, there is no visual indication of publication bias (Kendall tau=0.54, P=0.59).

For alcohol consumption in Figure 5, meta-analysis reveals a significant association in the random effect model (OR=1.42, 95% CI=1.23-1.65). A low statistical heterogeneity was shown among the ten case-control studies (I^2^=35%, P=0.13). The funnel plot did not show evidence of publication bias (Kendall tau=0.45, P=0.65). 

*Socioeconomic factors*

Tables 5 summarized the association of socioeconomic factors such as occupational exposure, wood dust exposure, dust exposure, and type of household with the risk of NPC. For occupational exposure, the only two case-control studies reported, suggested its association with NPC risk, and with both studies showing significant association between occupational exposure and NPC risk. For dust exposure, data obtained from the studies indicate non-significant association with NPC risk. For the type of household, five out of the six published studies found a significant association with NPC risk (Table 4). However, it was not possible to conduct a meta-analysis due to different types of the household definition used in each study. 

*Socio-demographic factors*

The association of NPC and sociodemographic factors such as education level were studied and summarized in Table 6. For education level, two (50%) out of the four case-control studies reported a significant association between education level and NPC risk. However, the summary effect was not computed using meta-analysis due to a different measure of education level in each study. 

## Discussion

Our meta-analysis summarizing the results from case-control studies suggests a statistically significant association of salted fish consumption with NPC risk. Our results also suggest a significant association of tobacco smoking and alcohol consumption with NPC risk, considering substantial heterogeneity across the studies. 

Salted fish consumption has significant association with NPC risk. Our Meta-analysis revealed significant association of salted fish to NPC risk with random effect model score showing OR of 1.41 at 95% confidence interval (CI) of 1.13-1.75 (P<0.01). Previous studies have demonstrated that the processes of preserving fish with salt triggers high nitrosamines accumulation which is carcinogenic (Chattopadhyay et al., 2017; Yong et al, 2017). Several foods such as salted fish and various preserved foods containing nitrosamines increase the incidence rate of NPC (Lo et al., 2004). In Southern China, an increase in NPC during childhood was significantly associated with salted fish consumption as reported by Zheng et al., (1994) in their previous work. An anthropological study survey conducted by Zheng et al., (1994), on living practices in high-NPC endemic regions and the association of preserved food consumption with NPC revealed that salted fish consumption significantly raises NPC risk with an odds ratio (OR) of 3.8 (p value=0.005) from the multivariate analysis (Zheng et al., 1994). Several case-control studies (Table 2) found a statistically significant association between salted fish consumption and NPC risk. These studies suggest that intake of salted fish could induce the development of NPC. Studies in many Asian countries reported that people who consume salted vegetables at least once a week are at higher NPC risk than those who rarely or never consumed such food (Yuan et al., 2000; Jia et al., 2010).

Previous studies have suggested that tobacco smoke contains high levels of nitrosamines, which have been associated with cancers, including NPC (Poirier et al., 1987; Yu et al., 1988). The association of smoking with NPC is related to squamous cells (Xue et al., 2013). Carcinogenic mechanism associated with tobacco smoke is initiated by direct contact of smoke with the epithelium of the nasopharynx, leading to direct action of the chemicals on the nasopharynx. Tobacco may also be contaminated with Epstein-Barr-virus-activating substances, which are significantly associated with undifferentiated nasopharyngeal cancer carcinoma (Jia and Qin, 2012).

The meta-analysis revealed that alcohol consumption was significantly associated to NPC risk with random effect model score showing OR: 1.42 at 95 % CI of 1.23 – 1.65. Alcohol is metabolized into acetaldehyde (AA) which adheres to proteins and DNAs to generate DNA carcinogenic adducts (Seitz and Mueller, 2015).

We also observed that consumption of salted vegetables and salted meat have significant association with the increased risk of NPC development (Table 2). This outcome is consistent with the previous studies conducted by Lee et al., (1994) (in Singapore) and Armstrong et al., (1998) (in Malaysia) which suggested that regular eating of salted vegetables can lead to increased risk of NPC (Lee et al., 1994; Armstrong et al., 1998). 

Our observation suggests that occupational exposure to wood dust was associated with increased risk of NPC. This result agrees with previous studies which have observed previously an association between wood exposure and nasal tumors (Loprieno, 1975; Vaughan and Davis, 1991). This association between NPC and wood can be attributed to chemicals such as chlorophenols used for wood preservation in wood industry (Hardell et al., 1982; Hardell et al., 1983).

The results for the association of the house type with NPC risk is significant according to our data. Several other reports have shown significant NPC association with different types of house (Hildesheim et al., 2001). Disparity in the measurements of the parameters studied in our sources of data limit the current meta-analysis. Overall, data collected in this study show some evidence that daily activities could increase the risk of developing NPC. However, this review did not discuss the molecular involvement of genes in the pathogenesis of NPC caused by these risk factors. 
